# Electroconvulsive therapy suppresses the neurotoxic branch of the kynurenine pathway in treatment-resistant depressed patients

**DOI:** 10.1186/s12974-016-0517-7

**Published:** 2016-02-29

**Authors:** Lilly Schwieler, Martin Samuelsson, Mark A. Frye, Maria Bhat, Ina Schuppe-Koistinen, Oscar Jungholm, Anette G. Johansson, Mikael Landén, Carl M. Sellgren, Sophie Erhardt

**Affiliations:** Department of Physiology and Pharmacology, Karolinska Institutet, SE-171 77 Stockholm, Sweden; Department of Clinical and Experimental Medicine, Section of Psychiatry, Faculty of Health Sciences, Linköping University, Linköping, Sweden; Department of Psychiatry & Psychology, Mayo Clinic College of Medicine, Rochester, MN USA; Protein Biomarkers, Personalized Healthcare & Biomarker Laboratories, Innovative Medicines, AstraZeneca, Gothenburg Sweden; Department of Clinical Neuroscience, Karolinska Institutet, Stockholm, Sweden; Science for Life Laboratory, Proteomics & Nanobiotechn, Karolinska Institutet, Stockholm, Sweden; Institute of Neuroscience and Physiology, The Sahlgrenska Academy at Gothenburg University, Gothenburg, Sweden; Department of Medical Epidemiology and Biostatistics, Karolinska Institutet, Stockholm, Sweden; Stanley Center for Psychiatric Research, Broad Institute of MIT and Harvard, Cambridge, MA USA; Department of Psychiatry, Massachusetts General Hospital, Harvard Medical School, Boston, MA USA

**Keywords:** NMDA receptor, Cytokines, ECT, Quinolinic acid, Inflammation, IL-6, Kynurenic acid, Treatment-resistant depression

## Abstract

**Background:**

Neuroinflammation is increasingly recognized as contributing to the pathogenesis of depression. Key inflammatory markers as well as kynurenic acid (KYNA) and quinolinic acid (QUIN), both tryptophan metabolites, have been associated with depressive symptoms and suicidality. The aim of the present study is to investigate the peripheral concentration of cytokines and tryptophan and kynurenine metabolites in patients with unipolar treatment-resistant depression before and after electroconvulsive therapy (ECT), the most effective treatment for depression.

**Methods:**

Cytokines in plasma from patients with major depressive disorder (MDD; *n* = 19) and healthy volunteers (*n* = 14) were analyzed with electrochemiluminescence detection. Tryptophan and kynurenine metabolites were detected with high-performance liquid chromatography (HPLC) and LC/MS. KYNA was analyzed in a second healthy control cohort (*n* = 22).

**Results:**

Patients with MDD had increased plasma levels of interleukin (IL)-6 compared to healthy volunteers (*P* < 0.05). We also found an altered kynurenine metabolism in these patients displayed by decreased plasma levels of KYNA (*P* < 0.0001) as well as a significantly increased QUIN/KYNA ratio (*P* < 0.001). Plasma levels of tryptophan, kynurenine, and QUIN did not differ between patients and controls. Treatment with ECT was associated with a significant decrease in the plasma levels of tryptophan (*P* < 0.05), kynurenine (*P* < 0.01), and QUIN (*P* < 0.001), whereas plasma levels of KYNA did not change. The QUIN/KYNA ratio was found to significantly decrease in ECT-treated patients (*P* < 0.05). There was a significant inverse correlation between symptom severity and kynurenine levels at baseline (*r* = −0.67, *P* = 0.002).

**Conclusions:**

This study confirms an imbalanced kynurenine pathway in MDD supporting the hypothesis of a netstimulation of *N*-methyl-d-aspartic acid (NMDA) receptors in the disorder. Treatment with ECT profoundly decreased QUIN, an NMDA-receptor agonist previously suggested to be implicated in the pathogenesis of depression, an effect that might have bearing for the good clinical outcome of ECT.

## Background

Major depressive disorder is a severe, life-threatening psychiatric illness, exposing the patient to direct risks, such as increased mortality due to cardiovascular disease, stroke, and alcohol abuse disorders. It is a leading cause of disability affecting millions of people worldwide and often causing chronic recurrent symptoms and profound socioeconomic burden [1]. With depression also follows a high risk of suicidal behavior, including attempts and completed suicides. More than 800,000 people die due to suicide every year, and it is estimated that for each adult who dies of suicide, there may have been more than 20 others attempting suicide [2]. Depression has been ranked as the fourth leading cause of disability worldwide by the World Health Organization [3], and the lifetime prevalence in high-income countries is 14 % [4].

The etiology of depression is still poorly understood, but recent hypotheses suggest that its development involves the activation of the innate immune system as well as alterations in glutamate excitotoxicity [5–7]. Indeed, increased concentrations of pro-inflammatory cytokines, chemokines, and acute-phase proteins have been observed in depressed patients. Increased circulating levels of interleukin (IL)-6 and C-reactive protein are the most frequently observed findings [8–10], but also, increased central levels of IL-6 have been described [11, 12].

Although evidence of immune activation in depression is emerging, the functional relationship to aberrant brain neurotransmission and behavior still needs to be established. Recently, activation of the kynurenine pathway has been suggested as a specific pathogenic mechanism that can explain this link [13–16]. Tryptophan degradation along this pathway produces several neuroactive compounds, such as quinolinic acid (QUIN), an *N*-methyl-d-aspartic acid (NMDA)-receptor agonist, and kynurenic acid (KYNA), a blocker of the NMDA receptor and the alpha7* nicotinic receptor (α7nAChR) [17]. Importantly, pro-inflammatory cytokines, especially interferon-γ but also IL-1β and IL-6, are potent inducers of indoleamine 2,3 dioxygenase (IDO-1) and tryptophan 2,3-dioxygenase (TDO2), the two main enzymes regulating the first step of the kynurenine pathway [18–21]. We, and others, have previously shown that KYNA is elevated in the cerebrospinal fluid (CSF) and postmortem brain of patients with psychotic disorders, such as schizophrenia and bipolar disorder [22–28]. With regard to depression, excessive NMDA-receptor signaling, tentatively through an increased QUIN/KYNA ratio, has been suggested [14, 15]. Thus, in a recent study, we found that CSF levels of QUIN were elevated in suicide attempters, and approximately half of these patients suffered from major depression. CSF QUIN was also correlated to CSF IL-6, indicating that inflammation had triggered the synthesis of QUIN [14]. Enhanced QUIN density has also been observed in the anterior cingulate gyrus of suicide victims [29]. Studies of peripheral kynurenines in depression are not totally uniform, but similar to CSF studies, they point to an overweight of the toxic branch of the kynurenine pathway. Thus, decreased plasma levels of KYNA [30, 31] and reduced plasma KYNA/QUIN and/or KYNA/3-hydroxykynurenine (3-HK) ratios [32, 33] have been described in depressed patients.

Since the 1950s, more or less all medications used for depression target the monoaminergic system. The efficacy of these medications is limited, and a substantial proportion of patients experience residual symptoms and persistent functional impairments or do not even respond to treatment [34]. Typically, they also have a lag time of several weeks [35], which is not satisfactory for acute suicide ideation and severe depression. Therefore, there is a major need to develop more effective therapies to treat these disorders and/or their deleterious phases by exploring other therapeutic approaches. An alternative treatment for more severe major depression, treatment-resistant depression, or suicidality is electroconvulsive therapy (ECT). This therapy triggers a brief seizure, by applying electric currents that pass through the brain.

Despite the longstanding use of ECT, the exact neurobiological mechanisms underlying its efficacy are not yet understood. Studies have primarily focused on the changes in neurophysiology [36], neuroendocrine [37], and neurogenesis [38] after ECT treatment. However, only a limited number of studies have addressed functioning of the immune system in relation to ECT for depression.

The aims of the present study are to analyze cytokines and kynurenines in treatment-resistant patients with major depressive disorder (MDD) and healthy controls and to relate these biomarkers to depression scores. We also investigate whether ECT influences cytokines and kynurenines in these patients.

## Methods

### Subjects

#### Patients

Nineteen patients diagnosed with MDD (11 males and 8 females) and prescribed ECT by their treating psychiatrist were included in the study. Venipuncture failed in three patients at blood sampling after the third ECT administration, and one patient was excluded due to upper respiratory tract infection at the time of ECT treatment. Data from these patients are included only at baseline. The diagnosis was established by a psychiatrist using the complete clinical presentation and a Mini-International Neuropsychiatric Interview (MINI) [39] or by two psychiatrists using the Montgomery-Åsberg Depression Rating Scale (MADRS) [40]. MADRS was available for 18 patients at baseline and for 15 patients after three ECT. Patients under the age of 18, involuntarily committed, subjected to ECT within 3 months prior to the study, or not able to understand the verbal and written information were excluded from the study. Eighteen patients had been adequately treated with oral antidepressant but had not responded, and one was treatment naïve. The concomitant drugs given were lithium (*n* = 1), antipsychotics (*n* = 3), benzodiazepines (*n* = 8), and hypnotic drugs (*n* = 12).

#### Healthy volunteers and healthy controls

##### Cohort 1

Fourteen male volunteers were mainly recruited among medical students, hospital staff members, and their relatives. They were all in good physical health and subjected to a medical check-up including laboratory tests (electrolytes, blood, thyroid, kidney, and liver) and a physical examination. All volunteers were free from medication for at least 1 month and free from any form of substance abuse, except for smoking (three healthy volunteers were smokers). Volunteers were subjected to a semi-structured interview using the Structured Clinical Interview for DSM-IV Disorders [41] or interviewed by a psychiatrist to exclude mental illness. None of them had a family history of major psychosis, major depression, or suicide in first- or second-degree relatives, and they were all found to be free from current signs of psychiatric morbidity or difficulties in social adjustment at the time of sampling.

##### Cohort 2

Population-based healthy controls were randomly selected by Statistics Sweden (SCB) and contacted by mail with a request for them to contact the study nurse if interested in participating in the study. Following a preliminary telephone screening, eligible persons were interviewed by experienced psychiatrists. Apart from psychiatric disorders and drug abuse, other excluded diagnoses were neurological conditions other than mild migraines, untreated endocrinological disorders, autoimmune disorders, pregnancy, and a family history of schizophrenia or bipolar disorder in first-degree relatives. Additionally for this study, individuals who had inflammatory disorders were excluded. Twenty-two such healthy controls were selected to match the probands, based on sex and age.

### ECT and plasma sampling

Patients were treated with ECT in the morning after at least 6 h of fasting at the University Hospital, Linköping, and at the Department of Clinical and Experimental Medicine, Division of Psychiatry, Faculty of Health Sciences, Linkoping University, Sweden. All patients received atropine before anesthesia was induced with thiopental. Muscle relaxation was induced with succinylcholine. The patients were ventilated with 100 % oxygen. A Mecta spectrum 5000Q device was used to give right unilateral ECT treatments with ultrabrief or brief pulse waves, and dosage was adjusted by age, sex, and outcome. Electroencephalography and a stopwatch were used to monitor the seizures. All plasma samples were collected between 8 and 11 am and after a night of fasting.

### Policy and ethics

This study was conducted in accordance with the latest Helsinki Protocol. The Ethics Committee of the Medical Faculty of Linköping University, Sweden; the Swedish Medical Products Agency; the Regional Ethics Committee in Stockholm; and the Swedish Data Inspection Board approved the study. All patients and healthy volunteers included received verbal and written information and gave their written informed consent. AstraZeneca did neither influence nor sponsor the clinical research performed at Linköping University or Karolinska Institutet. These data arise from a sample collection, not from a prospective trial, and is therefore not recorded in any clinical trial registry.

### Biochemical analyses

Blood samples were collected in heparinized tubes from overnight-fasting healthy volunteers, healthy controls, and patients. In patients, blood samples were collected before the first ECT and after the third ECT. Blood samples were centrifuged at (1438×*g*, 10 min), and plasma was collected and stored at −70 °C until analysis. All tryptophan metabolites were successfully detected in all plasma samples with an exception for KYNA and kynurenine not detected in one sample. In the second cohort of controls, only KYNA was analyzed.

#### Analysis of KYNA

Plasma samples were centrifuged at 21,000×*g*, for 5 min after deproteination with an equal volume of perchloric acid (0.4 M, containing 0.1 % sodium metabisulfite and 0.05 % EDTA). The centrifugation procedure was repeated followed by an additional 70 % perchloric acid (10 % by volume). Analysis of KYNA was measured by high-performance liquid chromatography (HPLC) with fluorescence detection, and the method has been outlined in detail previously [26]. Briefly, 50 μL of the deproteinized supernatant was injected to a ReproSil-Pur C18 reversed-phase column (4 × 150 mm; Dr. Maisch GmbH, Ammer-buch, Germany) using a fluorescence detector (Jasco Ltd., Hachioji City, Japan) with an excitation wavelength of 344 nm and emission wavelength of 398 nm (18-nm bandwith). A mobile phase containing 50 mM sodium acetate (pH 6.2) and 7 % acetonitrile was pumped through the reverse-phase column at a flow rate of 0.5 mL/min. Zinc acetate (0.5 M) was delivered post-columnar by a Pharmacia P-500 (GE Healthcare, Uppsala, Sweden) at a flow rate of 10 mL/h. The retention time of KYNA was approximately 7.5 min and was clear from other peaks with an exception for one sample that could not be distinguished due to an unknown peak at the same retention time. The sensitivity of the system was verified by analysis of a standard mixture of KYNA with concentrations from 0.25 to 30 nM, which resulted in a linear standard plot. The lower detection limit of the system was set to 0.625 nM. Some samples were analyzed in duplicates and confirmed that the intra-individual variation did not exceed 5 %.

#### Analysis of tryptophan, kynurenine, and QUIN

Plasma and standard samples (100 μL) were diluted two times with internal standard solution in 5 % formic acid and filtered at 2500×*g* for 60 min at 10 °C using 10-kDa Ultracel®-10 filter plates from Millipore. Internal Standards (IS) were added to each standard and plasma sample. Metabolites were purchased from Sigma-Aldrich (QUIN, kynurenine, and ^2^H_5_-tryptophan) and Synfine Research Inc., Ontario, Canada (^13^C_3_^15^N_1_-QUIN).

After centrifugation, 7.5 μL of the filtrate was injected using a Waters Acquity HPLC system equipped with a SymmetryShield™ RP18 2.1 × 100 mm, 3.5-μm particle column. The detection was performed using a Waters Xevo TQ triple quadrupole mass spectrometer operating in positive ionization MS/MS configuration. The mobile phase was run at a flow rate of 300 μL/min and consisted of 2.1 % formic acid in MilliQ water (A phase) and acetonitrile (B phase) starting with 5 % acetonitrile for 2 min following gradient elution, total run time of 10 min. The formic acid and acetonitrile were purchased as MS-grade from Sigma-Aldrich. The mass spectrometer was tuned for each analyte and set at a capillary voltage of 3.0 V, cone voltage of 16 V, source temperature of 150 °C, desolvation temperature of 350 °C, and desolvation gas flow of 650 L/h. Mass spectral transition for QUIN was m/z 168 > 106, kynurenine 209 > 146, and tryptophan 205 > 118 using collision energies of 14, 18, and 24 eV, respectively. Retention times for QUIN, kynurenine, and tryptophan were 1.2, 1.7, and 3.6 min, respectively.

#### Cytokine analyses

IL-1β, IL-2, IL-6, IL-8, IL-10, IL-12p70, tumor necrosis factor (TNF)-α, interferon (IFN)-γ, and granulocyte-macrophage colony-stimulating factor (GM-CSF) were quantified in plasma using a customized Human Ultra-Sensitive 9-Plex Kit (MesoScale Discovery, Gaithersburg, MD, USA) in 2013. The assays were analyzed as per the manufacturers’ protocol (http://www.mesoscale.com). Intra-assay coefficient of variation was below 25 % for all cytokines presented. The limits of detection (LOD) were IL-1β (0.36 pg/mL), IL-2 (0.35 pg/mL), IL-6 (0.27 pg/mL), IL-8 (0.09 pg/mL), IL-10 (0.21 pg/mL), IL-12p70 (1.4 pg/mL), TNF-α, (0.5 pg/mL), IFN-γ (0.74 pg/mL), and GM-CSF (0.20 pg/mL).

### Statistical analysis

All values are given as median and with interquartile range (IQR). Nonparametric tests were used due to a small sample size in each group (D’Agostino & Pearson omnibus normality test). Differences regarding plasma levels of cytokines and kynurenine metabolites between healthy controls and patients with depression were established using Mann-Whitney *U* test. Wilcoxon matched-pairs signed-rank test was used when computing differences in MADRS scores, plasma concentrations of cytokines, and kynurenine metabolites in patients before and after treatment with ECT. All correlation analyses were performed using a Spearman rank correlation analysis. Significance was assumed for all values where *P* < 0.05. All reported *P* values are two sided, and all analyses were corrected for age and gender when appropriate. The analyses were done using SPSS® Statistics 20.0 (IBM Inc., Chicago, IL, USA) or Prism® 6.0 (GraphPad Software Inc., La Jolla, CA).

## Results

### Demographics

In the present study, 19 patients (11 males and 8 females), 14 (all males) healthy volunteers, and 22 healthy controls (13 males and 9 females) were included. The median age was significantly higher in patients (41.0 years, IQR 25.0–54.0 years) than in healthy volunteers (24.0 years, IQR 23.0–26 years, *P* < 0.004, Mann-Whitney *U* test) but similar to the healthy controls (40.5 years, IQR 34.8–60.5 years). Patients were clinically evaluated with MADRS before ECT, and the median MADRS score for these patients was 37.0 (IQR 28.8–44.3). Following three ECT administrations, MADRS significantly decreased to 20.5 (IQR 14.0–26.5, *P* < 0.001).

### Cytokines in patients with MDD and healthy male volunteers

**Fig. 1:**
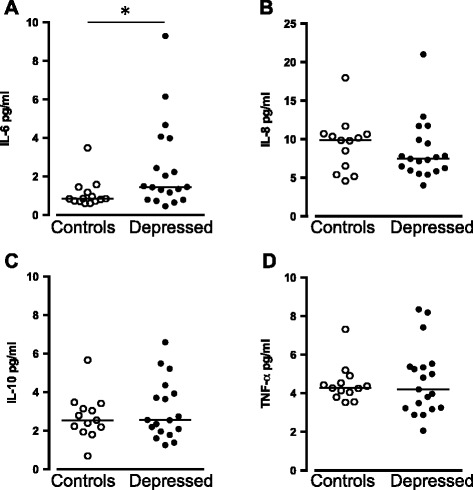
IL-6 (**a**), IL-8 (**b**), IL-10 (**c**), and tumor necrosis factor-α (TNF-α) (**d**) in the plasma of patients with MDD (*n* = 18–19) and controls (*n* = 13). Each *point* represents the concentration of a single plasma sample and the *horizontal lines* the median for each group. *P < 0.05 (Mann-Whitney *U* test, reported *P* value are two sided)

Plasma levels of IL-6, IL-8, IL-10, and TNF-α were detected in 13 of the 14 healthy male volunteers and in all 19 unipolar depressed patients with an exception for IL-10 and IL-6 that was not detected in one patient with MDD. The other cytokines analyzed were undetectable (IL-2) or found to be very close to the detection limit of the assay. Thus, IL-1β, IFN-γ, GM-CSF, and IL-12p70 were only detected in a limited number of samples and therefore not further analyzed. Plasma IL-6 concentration was elevated in patients with MDD (1.46 pg/mL, IQR 0.80–4.0 pg/mL, *n* = 18) compared to healthy male volunteers (0.85 pg/mL, IQR 0.72–1.25 pg/mL, *n* = 13; *P* = 0.029, Fig. 1a). When stratifying the analysis by sex, IL-6 was still significantly increased in male patients with MDD ([*n* = 11] 1.46 pg/mL, IQR 1.17–4.68 pg/mL vs. the healthy male volunteers [*n* = 13] 0.85 pg/mL, IQR 0.72–1.25 pg/mL; *P* = 0.0029). No correlation between age and IL-6 was observed in either patients (*ρ* = −0.45; *P* = 0.86) or in healthy male volunteers (*ρ* = −0.14; *P* = 0.63). We could not find any differences in the plasma levels of the other cytokines detected (IL-8, IL-10, and TNF-α) between patients with MDD and healthy male volunteers (Fig. 1b–d).

### Tryptophan and kynurenines in patients with MDD, healthy male volunteers, and healthy controls

**Fig. 2:**
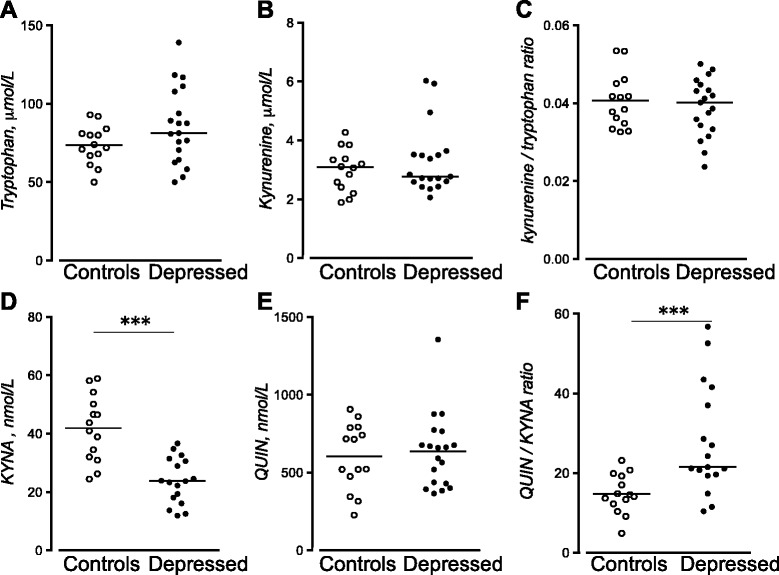
Tryptophan (**a**), kynurenine (**b**), kynurenine/tryptophan ratio (**c**), kynurenic acid (*KYNA*) (**d**), quinolinic acid (*QUIN*) (**e**), and QUIN/KYNA ratio (**f**), in the plasma of healthy controls (*n* = 14) and patients with MDD (*n* = 19). Each *point* represents the concentration of a single plasma sample and the *horizontal lines* the median for each group. ***P < 0.001 (Mann-Whitney *U* test) (all reported *P* values are two sided)

Plasma concentrations of KYNA were markedly decreased in patients with MDD (23.8 nM, IQR 17.6–31.8 nmol/L, *n* = 18) compared to KYNA levels in healthy male volunteers (42.4 nmol/L, IQR 31.8–51.2 nmol/L, *n* = 14; *P* < 0.0001, *P* [adjusted for age] = 0.001, Fig. 2d). An increased QUIN/KYNA ratio in MDD (22.9, IQR 19.6–40.1, *n* = 18) compared to healthy volunteers was also observed (14.4, IQR 11.8–19.4, *n* = 14; *P* < 0.001, *P* [adjusted for age] = 0.013, Fig. 2f). In patients and in healthy controls, we did not observe an effect of sex on either KYNA levels (*P* = 0.48 and *P* = 0.66, respectively) or the QUIN/KYNA ratio (*P* = 0.38, only patients).

No differences in the plasma levels of tryptophan or any of the other kynurenine metabolites (kynurenine, QUIN, or the ratio of kynurenine/tryptophan) were detected between patients with MDD and healthy volunteers (Fig. 2). Since the first control cohort included only male volunteers and were significantly younger than patients, plasma KYNA was measured in an age- and gender-matched second cohort of controls. Plasma KYNA was also here found to be significantly lower in patients with MDD. Plasma KYNA levels in the second cohort of controls were 40.6 nmol/L, IQR 33.0–54.8 nmol/L (*n* = 22; *P* < 0.0001).

### Tryptophan metabolites and cytokines after treatment with ECT in patients with MDD

**Fig. 3:**
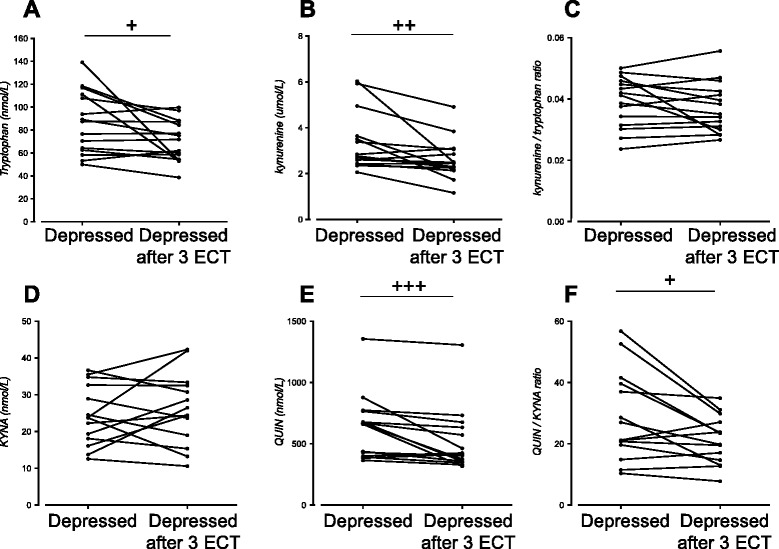
Tryptophan (**a**), kynurenine (**b**), kynurenine/tryptophan ratio (**c**), kynurenic acid (*KYNA*) (**d**), quinolinic acid (*QUIN*) (**e**), and QUIN/KYNA ratio (**f**), in the plasma from unipolar depressed patients before and after three ECT treatments (*n* = 15). ^+^ *P* < 0.0.5; ^++^ *P* < 0.01; ^+++^ *P* < 0.001 (Wilcoxon matched-pairs signed-rank test, reported *P* values are two sided)

Analysis of plasma tryptophan and kynurenine metabolites after treatment with ECT in patients with MDD revealed that plasma KYNA levels did not change but that plasma levels of tryptophan, kynurenine, and QUIN significantly decreased after three ECT (Fig. 3a, b, and e.) The QUIN/KYNA ratio significantly decreased after three ECT treatments (*P* < 0.05, see Fig. 3f).

The most pronounced effect of ECT was observed with regard to plasma QUIN levels, which was found to decrease in 80 % (12 out of 15) of the patients. No changes in cytokine levels were found after three ECT treatments (data not shown).

### Correlation between kynurenine and MADRS score in patients with MDD

**Fig. 4:**
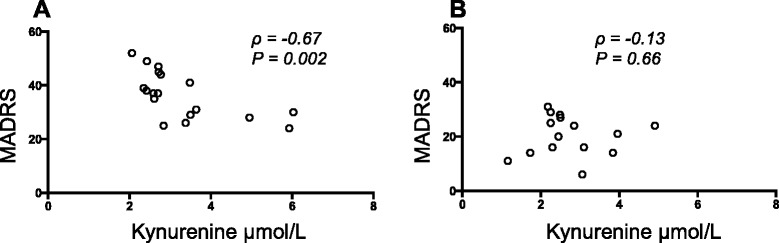
Correlation between plasma kynurenine and total scores from Montgomery-Åsberg Depression Rating Scale (*MADRAS*) before ECT treatment (**a**, *n* = 18) and after three ECT treatments (**b**, *n* = 15)

Plasma kynurenine was found to negatively correlate to MADRS score in patients with MDD before treatment with ECT (*ρ* = −0.67; *P* = 0.002, Fig. 4a). This correlation was not observed after three ECT administrations (*ρ* = −0.13; *P* = 0.66, Fig. 4b). Neither did tryptophan nor any other kynurenine metabolite or cytokine correlate to the MADRS score before or after ECT. We could not find any association between changes in the concentrations of tryptophan metabolites and the anti-depressive response (data not shown).

## Discussion

In the present study, we investigated the peripheral concentration of cytokines and kynurenine metabolites in unipolar patients diagnosed with an ongoing severe major depressive episode. Our data, showing increased plasma levels of IL-6, decreased KYNA levels, and an increased QUIN/KYNA ratio in these patients, supports the view of an ongoing low-grade inflammation [42] and an imbalanced kynurenine pathway [30–33] in MDD. The finding that the QUIN/KYNA ratio was increased in patients supports the hypothesis of a netstimulation of NMDA receptors in depression [30–33]. The observation of low plasma KYNA was confirmed in a second, age- and gender-matched, healthy control cohort. Furthermore, we found that treatment with ECT induced major changes in peripheral kynurenine metabolites in patients with MDD. Thus, plasma levels of tryptophan, kynurenine, and QUIN significantly decreased after a series of three ECT administrations whereas plasma levels of KYNA did not change. Interestingly, following three ECT, plasma QUIN levels were found decreased in 80 % of all patients. In addition, ECT was associated with a reduction in the QUIN/KYNA ratio, suggesting that NMDA receptors are to a lesser extent stimulated. In line with these findings, reduction in both CSF QUIN levels and in the CSF QUIN/KYNA ratio has previously been observed in suicide attempters following improvement of their depressive symptoms [14].

In the present study, we found increased plasma levels of IL-6 in patients with MDD. These results are in line with other studies showing increased levels of IL-6 in patients with depression [8, 12, 43]. It is well known that pro-inflammatory cytokines, including IL-6, can induce IDO, the rate-limiting enzyme of the kynurenine pathway. Thus, injection of IL-6 into rat hippocampus is associated with the induction of IDO [19] and fetal human cortical astrocytes respond to application of IL-6 with an increased KYNA synthesis [20]. In line with this, we recently found significantly positive correlation between IL-6 and kynurenine metabolites in CSF, both in suicidal attempters [14] as well as in patients with schizophrenia [20], suggesting an intimate interplay between this cytokine and the kynurenine pathway in the central nervous system. However, in the present study, such a correlation was not observed, indicating that this mechanism might be brain specific.

In line with previous studies, we also here found low levels of plasma KYNA in patients with MDD. Since recent studies show that age and gender influence plasma KYNA levels [44, 45], we included a second cohort of age- and gender-matched healthy controls. Also compared to this group, plasma KYNA was significantly decreased in patients with MDD. A challenging question is why peripheral KYNA is decreased in depressed patients. It has been suggested that a genetic variant in one of the enzymes catalyzing the final step in the synthesis of KYNA, kynurenine aminotransferase (KAT) III, is responsible [46]. Reduced expression or activity of the KAT III enzyme might thus lead to diminished synthesis of KYNA and consequently an imbalance between KYNA and QUIN [14, 15, 32, 33, 46]. It was recently reported that the total hippocampal and amygdala volume in patients with MDD correlated with a reduced plasma KYNA/QUIN ratio, indicating that an overweigh of the toxic branch of the kynurenine pathway may be of importance for the neurodegeneration occurring in these patients [33]. The ability of ECT to significantly decrease QUIN levels could thus be of clinical importance. Indeed, a recent longitudinal ECT study shows that repeated ECT administration increases the levels of KYNA, shifting the balance toward the neuroprotective branch of the kynurenine pathway. Thus, serum levels of KYNA and KYNA/3-HK increased over time with the most pronounced effect on KYNA after 3 months [47]. In the present study, we did not observe any increase in the plasma levels of KYNA, but since our study only lasted for 2 weeks, longitudinal changes cannot be excluded. Conceivably, the mechanism of action of ECT on patients with MDD could be due to its ability to abate NMDA-receptor stimulation by decreasing the levels of QUIN and/or strengthen NMDA-receptor antagonism by increasing the levels of KYNA. Interestingly, ketamine, an NMDA-receptor antagonist, has also been reported to exert a rapid antidepressant effect in patients with depression [48–50]. This effect of ketamine further suggests that depression could be directly associated with deficits in glutamatergic synaptic transmission.

In the present study, we also found a slight decrease of plasma tryptophan after repeated ECT treatments in line with a previous study [51]. Earlier studies, however, provide an inconsistent picture in this regard. Thus, unaltered peripheral levels of tryptophan have been reported in depressed patients after single or repeated ECT administrations [47, 52, 53], whereas two other studies showed increased plasma tryptophan levels after ECT [54, 55]. The reason for the variability between studies is not obvious but could be due to differences in tryptophan measured (free or total), number of ECT treatments, or time of sampling after the last ECT. It has also been suggested that anesthesia used during ECT could affect the plasma levels of tryptophan [53].

In line with a recent study [46], a negative correlation between plasma kynurenine and depression scores was observed. This might indicate that high levels of peripheral kynurenine are protective against depressive symptoms. Indeed, kynurenine can be effectively transported over the blood-brain barrier (BBB) through the neutral amino acid transporter. Approximately 60 % of brain kynurenine is coming from peripheral sources, and in the brain, kynurenine is transaminated to KYNA by the KAT enzymes in astrocytes or to QUIN by kynurenine 3-monooxygenase (KMO) in microglial cells. Compared to the higher capacity of KAT enzymes, displaying *K*_m_ values in the low milli-molar range [56], the KMO enzyme gets saturated at relatively low concentrations (*K*_m_ ≈ 20 μM) [57] and can therefore act as a rate-limiting step in the synthesis of QUIN. High levels of peripheral sources of kynurenine might, in this regard, rather contribute to increased levels of brain KYNA, which will guard against excessive NMDA-receptor stimulation and increase the threshold for depressive symptoms. In line with this hypothesis, we recently found that CSF KYNA was negatively associated with MADRS scores. Thus, the lower CSF KYNA, the worse degree of depression and suicidal symptoms [15]. In healthy volunteers, we recently detected a positive correlation between plasma kynurenine and CSF KYNA (unpublished observations), supporting the theory that the plasma levels of kynurenine observed in the present study reflect the central levels of KYNA in the patients.

In the present study, we observed that changes in the plasma QUIN levels associated with ECT treatment but not to the response of ECT as measured with MADRS. This is in line with a earlier study in depressed patients were CSF QUIN levels were found to correlate only to total scores on the Suicide Intent Scale but not to the mean score of MADRS or to the MADRS suicidality item [14]. This may indicate that QUIN does not affect depressive symptoms in general but rather has specific effects on suicidal behavior. This may also explain why we did not find any association between changes in the concentrations of QUIN after ECT and the anti-depressive response.

The results of the present study should be interpreted in light of its limitations. Firstly, we only had excess to plasma and not CSF, restricting our analysis to peripheral markers. Cytokines as well as the kynurenine metabolites, KYNA and QUIN, do not easily pass the BBB, and therefore, plasma levels of cytokines and kynurenines may not reflect changes in the brain. Secondly, for ethical reasons, all patients were on different drug treatments. Statistical corrections for drug treatment were not eligible in this small cohort of patients, and therefore, we cannot fully rule out that the pharmacological treatment did not influence contractions of cytokines or kynurenines.

## Conclusions

In conclusion, this study shows that ECT treatment reduces plasma QUIN levels in 80 % of the patients and that the treatment was associated with a reduction in the QUIN/KYNA ratio. Furthermore, we confirm previous studies showing decreased peripheral KYNA levels and an increased QUIN/KYNA ratio in patients, suggestive of a netstimulation of NMDA receptors in MDD. Our findings strengthen the hypothesis that kynurenine metabolites are key players in the pathophysiology of MDD and the capacity of ECT to decrease the neurotoxic branch of the kynurenine pathway might therefore be of clinical relevance.

## Abbreviations

3-HK: 3-hydroxykynurenine
BBB: blood-brain barrier
CSF: cerebrospinal fluid
ECT: electroconvulsive therapy
GM-CSF: granulocyte-macrophage colony-stimulating factor
IDO-1: indoleamine 2,3 dioxygenase
IFN-γ: interferon-γ
IL-6: interleukin-6
IQR: interquartile range
KAT: kynurenine aminotransferase
KMO: kynurenine 3-monooxygenase
KYNA: kynurenic acid
MADRS: Montgomery-Åsberg Depression Rating Scale
MDD: major depressive disorder
NMDA: *N*-methyl-d-aspartic acid
QUIN: quinolinic acid
TDO2: tryptophan 2,3-dioxygenase
TNF-α: tumor necrosis factor-α
α7nAChR: alpha7* nicotinic receptor
